# Editorial: Stereotactic body radiotherapy for prostate cancer

**DOI:** 10.3389/fonc.2022.1034987

**Published:** 2022-10-17

**Authors:** Amar U. Kishan, Joseph Kaminski, Filippo Alongi

**Affiliations:** ^1^ Department of Radiation Oncology, University of California, Los Angeles, Los Angeles, CA, United States; ^2^ Department of Urology, University of California, Los Angeles, Los Angeles, CA, United States; ^3^ Department of Radiation Oncology, University of Tennessee Health Science Center, Memphis, TN, United States; ^4^ Department of Radiology, Memphis Veterans Administration Medical Center, Memphis, TN, United States; ^5^ Advanced Radiation Oncology department, IRCCS Ospedale Sacro Cuore Don Calabria, Negrar dì Valpolicella (Verona), Verona, Italy; ^6^ Department of Radiation Oncology, University of Brescia, Verona, Italy

**Keywords:** prostate SBRT, prostate, active surveillance, spacer, MRI

Over the past few years, there has been an enormous growth in the strength of data suggesting the safety and efficacy of stereotactic body radiotherapy (SBRT) for prostate cancer. These include long-term data on ultrahypofractionated radiotherapy delivered with older techniques from the HYPO-RT-PC trial (1), early but robust data on modern SBRT from the PACE-B trial (2), and long-term follow-up data from a multi-institutional SBRT consortium (3). Further multi-institutional data (4) and several small randomized phase II trials (5, 6) have built a case for SBRT in high-risk prostate cancer and for use for metastasis-directed therapy in oligometastatic prostate cancer. Exciting clinical data also highlight the potential role for SBRT in the post-prostatectomy setting (7–10) and as a re-irradiation modality in radiorecurrent disease (11–13). Moreover, technological advances in real time image guided SBRT with MRI or PET have great potential. For example, the MIRAGE trial, the first randomized trial comparing MRI-guided with standard CT- guided SBRT seems to confirm a promising role for this innovative technique in prostate SBRT. (14) Yet, despite these advances, questions still remain. Can urinary and rectal toxicity be further mitigated? What patient factors—clinical, demographic, or otherwise—impact treatment efficacy? How can we better understand response to SBRT, both in the definitive setting and in the oligometastatic setting? And how does SBRT compare to other forms of re-irradiation?

This collection features 14 articles exploring the role of SBRT in prostate cancer across the entire spectrum of its natural history. Five manuscripts focus on practical considerations and interventions that might optimize the therapeutic ratio when delivering SBRT. Pham et al. explore the geometric distortions and variations in the urethra that might have dosimetric consequences for patients undergoing SBRT. Panizza et al. describe intrafraction motion during intact prostate SBRT as captured by electromagnetic tracking. Repka et al. review the rationale for using hydrogel spacers with prostate SBRT (Repka et al), while Kundu et al. provide a dosimetric and toxicity analysis of patients who received SBRT and either had or did not have a spacer placed. Finally, Greco et al. describe seven year outcomes following dose-escalated SBRT wherein an endorectal balloon was used for mobilization/stabilization and a Foley catheter with implanted electromagnetic beacons was used to track and spare the urethra.

The next set of six articles explore efficacy and toxicity profiles among patients receiving SBRT for localized prostate cancer. Fuller et al. provide long-awaited 10-year outcomes from a multi-center phase II trial of high dose rate brachytherapy-like SBRT for intermediate risk prostate cancer. Three reports from the Georgetown SBRT group explore the incidence and natural history of post-SBRT hematospermia (Shah et al.), the implications of treatment interruptions (Pepin et al.), and the financial burden of SBRT (Sholklapper et al.). Correa and Loblaw provide a detailed review of the clinical evidence for SBRT in the context of high-risk disease. Finally, Liu et al. describe the ARGOS/CLIMBER protocol, in which men have pre-treatment and scheduled post-treatment multiparametric MRI and prostate specific membrane antigen positron emission tomography to establish imaging biomarkers of treatment response.

The collection is rounded out by three articles focusing on patients with recurrent disease. Cuccia et al. report on the outcomes of MRI-guided SBRT for re-irradiation of the prostate in men with radiorecurrent disease. Ryg et al. similarly explore long-term outcomes following salvage high dose-rate brachytherapy vs. salvage CT-guided SBRT. Finally, Mercier et al. report survival outcomes and failure patterns among men getting metastasis-directed SBRT for hormone sensitive or castrate resistant metastatic prostate cancer.

SBRT has demonstrated great success thus far for the definitive treatment of localized prostate cancer, but multiple important areas of investigation exist. These include: optimizing treatment by minimizing toxicity, evaluating long-term results in low and intermediate risk disease, better understanding its role in high risk disease, developing better markers of radioresponsiveness, exploring SBRT in the post-prostatectomy setting and for re-irradiation of radiorecurrent disease, and optimizing SBRT for metastasis-directed therapy. We hope that this broad collection of articles summarizes these exciting areas of active research while underscoring the efficacy and safety of SBRT for prostate cancer.

## Author contributions

All authors listed have made a substantial, direct, and intellectual contribution to the work and approved it for publication.

## Conflict of interest

The authors declare that the research was conducted in the absence of any commercial or financial relationships that could be construed as a potential conflict of interest.

## Publisher’s note

All claims expressed in this article are solely those of the authors and do not necessarily represent those of their affiliated organizations, or those of the publisher, the editors and the reviewers. Any product that may be evaluated in this article, or claim that may be made by its manufacturer, is not guaranteed or endorsed by the publisher.
